# Effect of Magnetized Water Mouthrinse on *Streptococcus mutans* in Plaque and Saliva in Children: An *in vivo* Study

**DOI:** 10.5005/jp-journals-10005-1461

**Published:** 2017-02-27

**Authors:** Anil K Goyal, Ambika S Rathore, Mamta Garg, Rinku Mathur, Meenakshi Sharma, Abhishek Khairwa

**Affiliations:** 1 Assistant Professor, Department of Pediatric and Preventive Dentistry, Rajasthan University of Health Sciences, College of Dental Sciences, Jaipur Rajasthan, India; 2 Assistant Professor, Department of Pediatric and Preventive Dentistry, Rajasthan University of Health Sciences, College of Dental Sciences, Jaipur Rajasthan, India; 3 Private Practitioner, Department of General Dentistry, Goyal Child and Dental Clinic Jaipur, Rajasthan, India; 4 Assistant Professor, Department of Pediatric and Preventive Dentistry, Rajasthan University of Health Sciences, College of Dental Sciences, Jaipur Rajasthan, India; 5 Assistant Professor, Department of Pediatric and Preventive Dentistry, Rajasthan University of Health Sciences, College of Dental Sciences, Jaipur Rajasthan, India; 6 Senior Lecturer, Department of Pediatric and Preventive Dentistry, Jaipur Dental College, Jaipur, Rajasthan, India

**Keywords:** Antimicrobial efficacy, Magnetized water, Mouth-wash, Saliva, *Streptococcus mutans* plaque.

## Abstract

**Aim:**

This study was conducted to evaluate and compare the antimicrobial efficacy of magnetized water as a mouthwash on colony count of *Streptococcus mutans* in children.

**Materials and methods:**

Total sample size of 30 children were selected out of screened 290 children by simple random sampling between the age group of 7 and 12 years. The study was conducted over a period of 2 weeks. After selection of the children according to inclusion and exclusion criteria, children were allowed using 10 mL of 72 hours magnetized water for 3 minutes twice in a day for a period of 2 weeks, and further plaque and saliva samples were collected at 1- and 2-week intervals from baseline. Microbiological analysis of plaque and saliva samples was done by Dentocult SM strip kit (Orion Diagnostica, Finland), and the results were statistically analyzed and tabulated.

**Results:**

Statistically, there was highly significant reduction in S. *mutans* count in plaque as well as in saliva after 1- and 2-week intervals from baseline.

**Conclusion:**

So, finally our study showed that magnetized water is as effective a mouthwash against *S. mutans* and has better action in plaque as compared with saliva. It can be used as an adjunct to commercially available mouthwashes.

**How to cite this article:** Goyal AK, Rathore AS, Garg M Mathur R, Sharma M, Khairwa A. Effect of Magnetized Wate**i** Mouthrinse on *Streptococcus mutans* in Plaque and Saliva in Children: An *in vivo* Study. Int J Clin Pediatr Dent 2017 10(4):335-339.

## INTRODUCTION

Our body is over 70% water. All biological functions including circulation, digestion, absorption, and excretion depend on water. Water is required for blood, the lymphatic system, and for healthy skin and muscles. It is well known among health professionals that the pH of our body is more acidic when we are sick. Magnetized water, which is more alkaline, raises the pH of our body, which allows the body to get rid of the toxins. Bio-magnetized water is believed to be energy-building, activating, cleansing, and detoxifying.^1^

Water is paramagnetic—meaning that it will hold a magnetic charge. In nature, the earth’s magnetic field naturally charges water in lakes, wells, and running streams. However, as water passes through the treatment plants and is transported through pipes, it loses its magnetic charge. Treating water with magnetic fields simply restores the natural energy and balance that nature intended. Magnetized water has more hydroxyl (OH^-^) ions that form alkaline molecules which reduce the acidity. Normal tap water has a pH of about 7. Magnetized water is more alkaline and can have a pH as high as 9.2. Magnetizing water reduces the surface tension of the water making it feel softer. It is thinner, wetter, and more absorbable, so it is better able to penetrate cell walls and deliver the nutrients that it carries.^1^

Magnetism is well known in the field of physics. Magnets prove to be strong safeguard against illness and serve as a highly beneficial preventive device. It is known to have the power of drawing pain out from the body, of relieving stiffness of joints and muscles, and of removing toothache immediately.

When water passes through the magnetic field, it undergoes certain changes. The magnetic field alters the electrical characteristics of hydrogen ions as well as minerals.

The bio-south magnets help in reducing scale buildup. The bio-north magnetic water is used in the treatment of various diseases. The bio-south magnetic field stimulates the system.

The force of magnetism has a great influence on living organism. When a permanent magnet is kept in continuous contact with water, for considerable time, the water is not only influenced by the magnetic flux of magnet but also becomes magnetized and acquires magnetic properties. Such magnetized water has its effect on human body when taken internally, regularly for a considerable period. Best results are achieved when water is magnetically treated just prior to use.^2^

Biological effect of static magnetic field was investigated by using ferrite magnets to conduct a magnetic field exposure experiment on three species of bacteria: *S. mutans, Staphylococcus aureus,* and *Escherichia coli.* The results showed that the ferrite magnet caused strength-dependent decrease in the growth rate and growth of maximum number of bacteria for *S. mutans* and *S. aureus* when cultured under anaerobic conditions, but that their growth was not inhibited under aerobic conditions.^3^

Therefore, keeping in mind these facts about magnetized water, this study was conducted to evaluate the antimicrobial efficacy of conventionally prepared magnetized water as a mouthwash on colony count *S. mutans* in children.

## MATERIALS AND METHODS

Total sample size of 30 children was selected out of screened 290 children by simple random sampling between the age group of 7 and 12 years. The study was conducted over a period of 2 weeks.

The subjects volunteered to participate after verbal and written information. Ethical clearance was taken from the Ethical Clearance Committee and informed consent was taken from the parents of all the children.

The selected sampling was done taking into consideration the following criteria:

### Inclusion Criteria

- Systemically healthy patients
- No fixed or removable orthodontic appliances or removable prosthesis
- No use of magnetized water as oral rinse earlier
- No history of oral prophylaxis done for at least 3 months prior to study
- Mixed dentition period
- Decay-missing-filled (dmf) score >4

### Exclusion Criteria

- History of fluoride treatment in the past 2 weeks
- Eating before 1 to 2 hours before sample collection
- Use of antimicrobial mouthrinse before several hours of sample collection
- History of antibiotic therapy in the subjects within previous 3 months.

After the selection of the children according to inclusion and exclusion criteria, children were allowed using 10 mL of 72 hours magnetized water for 3 minutes twice in a day.

Oral prophylaxis of all the subjects was done using ultrasonic scaler. Then the subjects were instructed to abstain from any oral hygiene measures for next 24 hours.

### Method of magnetizing Water

Reverse osmosis water was taken in glass bottles and kept over the magnets for 72 hours for magnetization.

### Plaque Sample Collection

After 24 hours of oral prophylaxis, baseline samples were collected for both groups of subjects. Culture vials were taken at room temperature 1 hour prior to the sample collection. Bacitracin disks were placed in the culture vials 15 minutes before the sample collection and vials were shacked thoroughly.

Plaque samples were collected with a sterile probe tip from four specific sites including^4^:

- Buccal surface of right maxillary first molar
- Labial surface of maxillary incisor
- Lingual surface of mandibular incisor
- Lingual surface of the left mandibular first molar.

Collected plaque samples were evenly and thoroughly distributed on square tip plaque strip (Dentocult SM Strip Mutans Kit, Orion Diagnostica, Finland) using sterile ear buds. The same procedure was repeated after 1 and 2 weeks.

### Saliva Sample Collection

Before collection of salivary samples, the subjects were asked to chew paraffin tablets for 1 minute and then spit the remaining saliva. Saliva samples were collected by pressing the round tip salivary strips against the dorsal surface of tongue and removed with gently closed lips. The same sample collection procedure was repeated after 1 and 2 weeks.

After collection of both saliva and plaque samples, the strips were placed in the labeled culture vials and incubated at 37°C for 48 hours. After incubation the colony counts of *S. mutans* were interpreted using model chart provided by manufacturer and score were given from 0 to 3 after comparing the incubated strips with model chart.

After collecting the baseline samples, the subjects were given the mouthrinses and were asked to rinse under supervision for a period of 2 weeks.

The subjects were then asked to start maintaining their regular oral hygiene measures.

## STATISTICAL ANALYSIS

### Formula used for calculating Standard Deviation

Standard deviation is the most frequently used method of deviation. It is defined as root mean square deviation and is denoted by S or SD.

**Graph 1:**
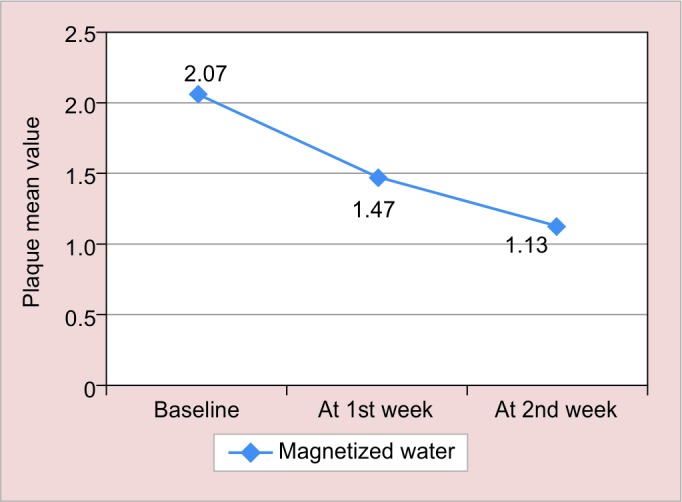
Mean + SD of S. *mutans* in magnetized water subjects from baseline to various intervals in plaque

**Graph 2:**
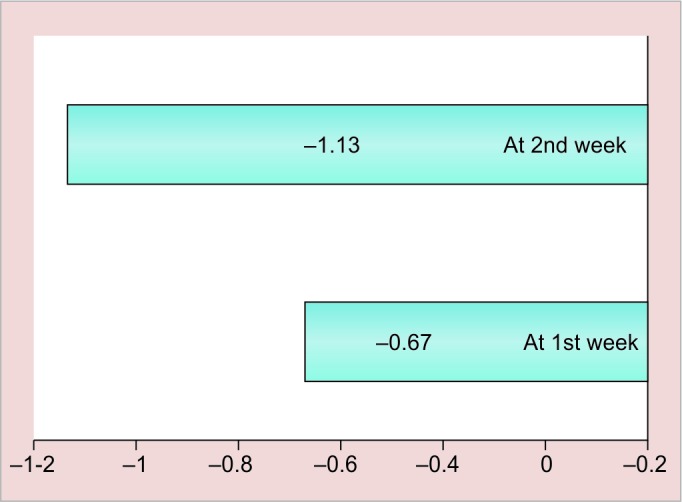
Mean change ± SD of S. *mutans* in magnetized water subjects from baseline to various intervals in plaque

**Graph 3:**
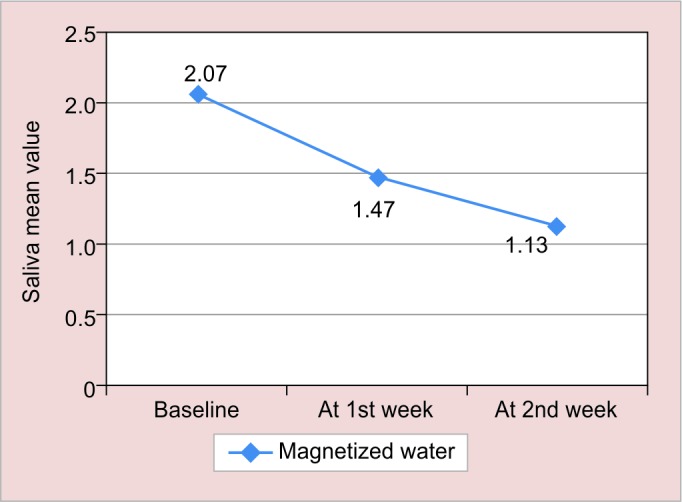
Mean ± SD of S. *mutans* in group I (magnetized water) subjects from baseline to various intervals in saliva

**Graph 4:**
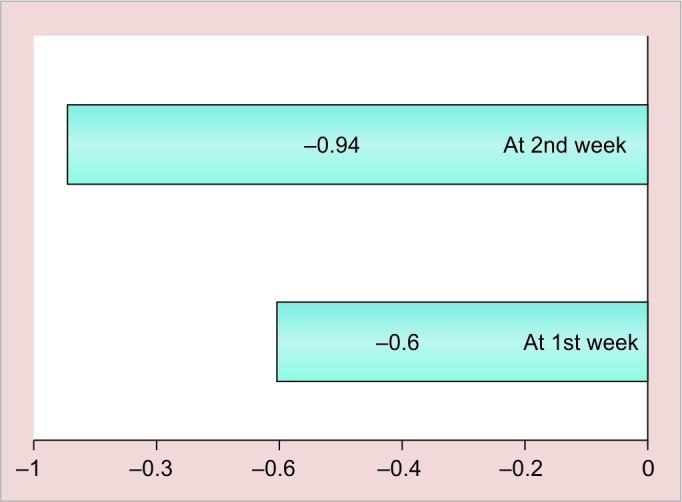
Mean change ± SD of S. *mutans* in group I (magnetized water) subjects from baseline to various intervals in saliva

## RESULTS

The results of this study are shown in Graphs 1 to 4 and tabulated in Tables 1 to 4.

## DISCUSSION

Wevangti Vangra^5^ reported that when water is magnetically charged, it electrically takes on a greater ionic charge than the minerals which create a natural magnetic attraction between the two. Softening and better taste occur from an actual reduction in size of water molecule. The small magnetized water molecule has a greater solvency and a magnetic attraction.

**Table 1:** **Table 1:** Mean ± SD of S. *mutans* in magnetized water subjects from baseline to various intervals in plaque

		*Interval*					
*Group*		*Baseline*		*At 1st week*		*At 2nd week*	
Magnetized water (plaque)		2.07 ± 0.44		1.47 ± 0.62		1.13 ± 0.72	

**Table 2:** **Table 2:** Mean change ± SD of S. *mutans* in magnetized water subjects from baseline to various intervals in plaque

		*Interval*			
		*At 1st week*		*At 2nd week*	
Mean change ± SD		0.60 ± 0.49		0.94 ± 0.57	
p-value		<0.001		<0.001	
Significance		HS		HS	

**Table 3:** **Table 3:** Mean ± SD of S. *mutans* in magnetized water subjects from baseline to various intervals in saliva

		*Interval*					
*Group*		*Baseline*		*At 1st week*		*At 2nd week*	
Magnetized water (saliva)		2.07 ± 0.44		1.47 ± 0.62		1.13 ± 0.72	

**Table 4:** **Table 4:** Mean change ± SD of S. *mutans* in magnetized water subjects from baseline to various intervals in saliva

		*Interval*			
		*At 1st week*		*At 2nd week*	
Mean change ± SD		0.60 ± 0.49		0.94 ± 0.57	
p-value		<0.001		<0.001	
Significance		HS		HS	

The magnetic field affects water molecules by:

- The molecules increase in size, which increases the water solubility and permeability (the ability to dispense and penetrate other substances). The increase in permeability assists in the dissolution of nutritional substances and improves the body adsorption of water as well as the nutritional substances. Also when the size of water molecule is increased, its ability to absorb toxins is much greater.
- Being slightly alkaline—Water ionization: When water is affected by electromagnetic vibrations, some molecules of water will separate to hydrogen ion (H+) and hydroxyl ion (OH^-^). Some hydroxyl ions will combine with minerals, such as calcium and become calcium bicarbonate which has an alkaline property. Magnetized water has pH value of about 7.6 to 8.5.
- Less surface tension—It reduces from 75 to 45 dynes or even 38 dynes because of microcluster. It also reduces blood viscosity.
- Microcluster—Magnetized water has a smaller group of molecules per water cluster than regular water. It has six molecules while regular water has 14 to 30. By this, it is easier to pass through cell membrane because of microcluster. It increases metabolism because it is easier to transport nutrients and oxygen to cell and to carry out toxins and waste matter from cell.
- Antioxidant—Some hydroxyl ions (OH^-^) combine together and become water and oxygen ion (O). This oxygen ion can stop free radical cycle because it is negatively charged ion. Free radical is any molecule that has a single unpaired electron in an outer shell. This free radical will steal electron from body, and will cause other molecule lose their electron. That molecule will steal electron from body, cause another free radical. This is called “free radical cycle.”
- Have lots of oxygen—When some oxygen ion will combine together and become oxygen, this oxygen can dissolve immediately in that water. If we put the magnetized water in closed bottle, there are small bubbles that get attached to the walls of bottle. It is said that, “Water which has alkaline property, always has oxygen inside.” This gives energy to the cells, prevents development of anaerobic bacteria, and stops their growth.

In our study subjects baseline value in plaque was 2.07 ± 0.44, which reduced after rinsing with magnetized water to 1.47 ± 0.62 and 1.13 ± 0.72 after 1 and 2 weeks respectively, which was also seen in a previous study done by Gupta and Bhat^6^ by using 72 hours magnetized water as mouthwash for a period of 1 week. They found the increase in the mean change at various levels, which signifies fall in *S. mutans.*

Lone et al^7^ did a study on magnetized water and chlorhexidine on plaque accumulation and gingival inflammation and found that chlorhexidine was marginally good on magnetized water.

Mean change and standard deviation of *S. mutans* count in plaque followed by mouth rinsing with magnetized water from baseline to various intervals in group I subjects after 1 week are calculated. The mean change from baseline value was 0.60 ± 0.49 and after 2 weeks it was 0.94 ± 0.57, which is statistically highly significant with respect to p-value that was <0.001 for both 1- and 2-week intervals from baseline, which shows that our results are concurrent with previous study done by Gupta and Bhat^6^ by using 72 hours magnetized water as a mouthrinse for a period of 1 week.

Another study was done by Kohno et al^3^ to evaluate the biological effect of static magnetic field using ferrite magnets to conduct a magnetic field exposure experiment on three species of bacteria: *S. mutans, S. aureus,* and *E. coli.* The effects were evaluated by culturing the bacteria and determining their growth rate, the maximum numbers of bacteria, and [3H]-thymidine incorporation. The results showed that the ferrite magnet caused strength-dependent decreases in the growth rate and growth of maximum number of bacteria for *S. mutans* and *S. aureus* when cultured under anaerobic conditions, but that their growth was not inhibited under aerobic conditions.

- *Wevangti Vangra^5^—water ionization:* when water is affected by electromagnetic vibrations, some molecules of water will separate to hydrogen ion (H+) and hydroxyl ion (OH^-^). Some hydroxyl ions will combine with minerals, such as calcium and become calcium bicarbonate which has alkaline property. Magnetized water has pH value of about 7.6 to 8.5.
- Antioxidant property in which some hydroxyl ions (OH^-^) combine together and become H_2_O and oxygen ion (O). This oxygen ion can stop free radical cycle because it is negatively ion.

When some oxygen ion will combine together and become oxygen, this oxygen can dissolve immediately in that water. If we put the magnetized water in closed bottle, there are small bubbles that get attached to the walls of bottle. It is said that, “Water which has alkaline property, always has oxygen inside.” This gives energy to the cells, prevents development of anaerobic bacteria, and stops their growth.

- The present study also demonstrate the same that since magnetized water is alkaline and also as *S. mutans* is anaerobic bacteria, therefore, its alkaline property stops the anaerobic bacteria to grow, thereby reducing the count.

Magnetohydrodynamics prevents naturally occurring minerals deposits in fluids, changing from liquid to a solid state.^8^ This occurs by interruption of the normal process of colonization (electrovalent bonding of cations) and therefore, prevents the formation of deposits which would otherwise adhere to a host surface. By this principle, the bonding process by which bacteria colonizes, and by which plaque and calculus adhere and accumulates on teeth is inhibited.

## CONCLUSION

So finally our study showed that magnetized water is as effective as a mouthwash against *S. mutans* and has better action in plaque as compared with saliva. It can be used as an adjunct to commercially available mouthwashes.

## References

[1] Forsythe J The miracle of biomagnetic water. Available from:. www.ArticleClick.com.

[2] Hari AR. Water—a miracle therapy magnetized water..

[3] Kohno M, Yamazaki M, Kimura I, Wada M (2000). Effect of static magnetic fields on bacteria: *Streptococcus mutans, Staphylococcus aureus,* and *Escherichia coli.*. Pathophysiology.

[4] Asokan S, Rathan J, Mutthu MS, Rathna PV, Emmadi P (2008). Raghuraman, Chamundeshwari. Effect of oil pulling on *Streptococcus mutans* count in plaque and saliva using Dentocult SM strip mutans test: a randomized controlled triple blind study.. J Indian Soc Pedod Prev Dent.

[5] Available from:. http://EzineArticles.com.

[6] Gupta N, Bhat M (2011). Comparative evaluation between 0.2% chlorhexidine mouth rinse and magnetized water rinse on *streptococcus mutans* in children.. Int J Clin Pediatr Dent.

[7] Lone N, Sidiq M, Khan M, Shah AF, Yousuf A (2016). Short term effects of magnetised water and chlorhexidine on plaque accumulation and gingival inflammation—a randomised clinical study.. Ann Int Med Dent Res.

[8] Hibben SO (1973). Magnetic treatment of water. National Technical Information Services no. AD-757 887..

